# Supplementary material for: Splice-Junction-Based Mapping of Alternative Isoforms in the Human Proteome
Source: Cell Rep. Author manuscript; Available in PMC 2020 Jan 15. (PMC6961840; doi:10.1016/j.celrep.2019.11.026)
Supplement: 2 [file NIHMS1546469-supplement-2.zip › DF1/PXD010154/Prostate/Prostate_17_CLINT1_CLILWARPR.pdf]

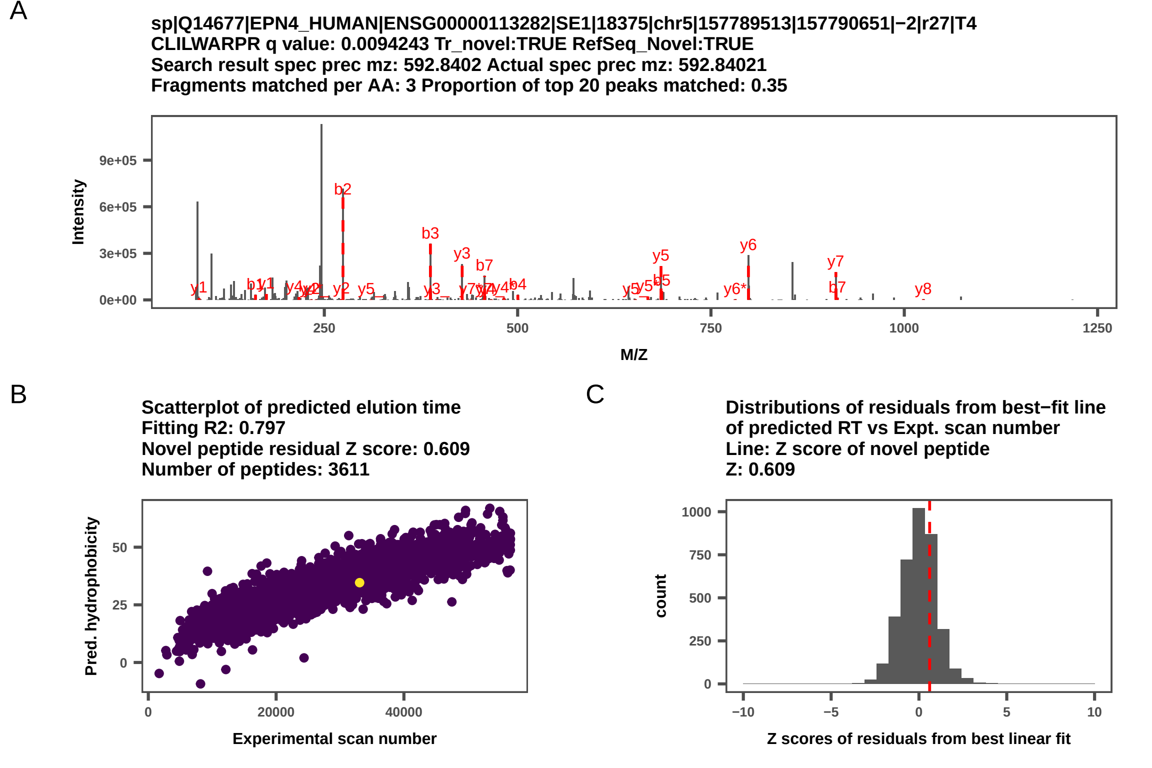
A
sp|Q14677|EPN4_HUMAN|ENSG00000113282|SE1|18375|chr5|157789513|157790651|−2|r27|T4
CLILWARPR q value: 0.0094243 Tr_novel:TRUE RefSeq_Novel:TRUE
Search result spec prec mz: 592.8402 Actual spec prec mz: 592.84021
Fragments matched per AA: 3 Proportion of top 20 peaks matched: 0.35
Intensity
9e+05
6e+05
3e+05
0e+00
250
500
750
1000
1250
M/Z
b2
b3
y3
b7
y5
y6
y7
y8
B
Scatterplot of predicted elution time
Fitting R2: 0.797
Novel peptide residual Z score: 0.609
Number of peptides: 3611
Pred. hydrophobicity
50
25
0
20000
40000
Experimental scan number
C
Distributions of residuals from best−fit line
of predicted RT vs Expt. scan number
Line: Z score of novel peptide
Z: 0.609
count
1000
750
500
250
−10
−5
5
10
Z scores of residuals from best linear fit
